# Therapeutic Benefits of Pomegranate Flower Extract: A Novel Effect That Reduces Oxidative Stress and Significantly Improves Diastolic Relaxation in Hyperglycemic In Vitro in Rats

**DOI:** 10.1155/2022/4158762

**Published:** 2022-06-08

**Authors:** Yuanyuan Wei, Ahmad Taha Khalaf, Peng Ye, Wei Fan, Junyi Su, Wanlu Chen, Hao Hu, Rashid Menhas, Lifeng Wang, Zahraa Oglah

**Affiliations:** ^1^College of Medicine, Chengdu University, Chengdu 610106, Sichuan, China; ^2^Department of Anatomy and Histology, College of Medicine, Chengdu University, Chengdu 610106, Sichuan, China; ^3^School of Pharmacy, Chengdu University of Traditional Chinese Medicine, Chengdu 610106, Sichuan, China; ^4^College of Medical, Fujian Medical University, Fuzhou 350108, Fujian, China; ^5^Research Center of Sport Social Sciences, School of Physical Education and Sports, Soochow University, Suzhou, Jiangsu, China; ^6^Department of Physiology, Xinjiang Medical University, Urumqi 830011, Xinjiang, China; ^7^School of Science, Auckland University of Technology (AUT), 55 Wellesley Street East, Auckland, New Zealand

## Abstract

The pomegranate flower is an ancient herb in traditional Chinese medicine with multiple properties. Recent studies have shown that pomegranate flower extract is beneficial, especially for hyperglycemia. In this experiment, we investigated the diastolic effect of pomegranate flower polyphenol (PFP) extract on the isolated thoracic aorta of rats in both the absence and presence of high glucose levels. Isotonic contractile forces were recorded from aortic rings (about 3 mm in length) from rats using the BL-420F Biological Function Test System. Tissues were precontracted with 60 mM KCl to obtain maximum tension under 1.0 g load for 1 hour before the balance was achieved, and the fluid was changed every 15 minutes. PFP (700 mg/L–900 mg/L) showed a concentration-dependent relaxant effect on the aortic rings; vasodilation in the endothelium-intact was significantly higher than that in the de-endothelialized segments (*P* < 0.01). The endothelium-dependent vasorelaxant effect of PFP was partially attenuated by K^+^ channel blockers, tetraethylammonium (TEA), glibenclamide (Glib), and BaCl_2_, as well as L-NAME (eNOS inhibitor) on the denuded endothelium artery ring. Concentration-dependent inhibition of PFP on releasing intracellular Ca^2+^ in the Ca^2+^-free solution and vasoconstriction of CaCl_2_ in Ca^2+^-free buffer plus K^+^ (60 mM) was observed. In addition, PFP (0.1–10 mg/L) showed significant inhibition of acetylcholine-induced endothelial-dependent relaxation in the aorta of rats in the presence of high glucose (44 mmol/L). Nevertheless, the vasodilating effect of PFP was inhibited by atropine and L-NAME. The results indicated that PFP-induced vasodilation was most likely related to the antioxidant effects through enhanced NO synthesis, as well as the blocking of K^+^ channels and inhibition of extracellular Ca^2+^ entry. In conclusion, these observations showed that PFP ameliorates vasodilation in hyperglycemic rats. Hence, our results suggest that PFP supplementation may be beneficial for hypertensive patients with diabetes.

## 1. Introduction

In traditional Chinese medicine, the pomegranate flower has several medicinal uses, including as an antidiarrheal drug and as an astringent agent [1]. Our previous studies also showed that it is useful in treating hyperglycemia and dyslipidemia [2, 3]. However, few studies reported its effect on the function of blood vessels. Vasodilation dysfunction is the major pathological change of blood vessels in diabetes mellitus and the pathological basis of diabetic vascular complications, as well as atherosclerosis and other cardiovascular diseases [4, 5]. The endothelium plays a crucial role in regulating the tone of vascular smooth muscle by releasing dilator and constrictor substances, such as nitric oxide [6, 7]. Nonendothelium-dependent vasodilatation is mainly mediated by cytoplasmic Ca^2+^ levels and potassium channels that importantly contribute to the vasomotion of vascular smooth muscle (VSM) by regulating the open-state probability of voltage-gated Ca^2+^ channels (VGCC) [8–10].

Our preliminary study showed that pomegranate flower polyphenol (PFP) extract is an effective antioxidant that possesses hypoglycemic effects and anti-inflammatory properties [11]. In addition, PFP exhibits vasodilation effects on vascular endothelium in diabetic rats through inhibiting endothelial-derived contracting factors, such as endothelin-1, angiotensin II, and thromboxane B_2_, and increasing 6-keto-PGF1a concentrations in plasma [12]. In the present study, we aimed to investigate the mechanisms of the vasorelaxation effect of PFP using isolated aortic rings from rats.

## 2. Materials and Methods

### 2.1. Animals and Ethical Statement

The study was carried out in accordance with the “Guide for the Care and Use of Laboratory Animals,” published by the US National Institutes of Health, 2013/63/EU, for animal experiments. The present protocol was approved by the local ethics committee (Experimental Animal Centre of Xinjiang Medical University). All efforts were made to minimize animal suffering and reduce the number of animals used. Specific pathogen-free SPF-grade male Sprague Dawley rats (250–300 g) were supplied by the Experimental Animal Centre of Xinjiang Medical University (license no. SCXK (Xin) 2018–0004).

### 2.2. Preparation of PFP and Total Phenolics Measurement

Fresh pomegranate flowers were collected in Hotan, Xinjiang, China, identified by experts, and then cleaned and crushed. PFP was a commercial, spray-dried ethanol extract without an excipient. The extraction of dried pomegranate flower powder involved using 70% ethanol for 20 hours at 95°C. The resulting extract was filtered, concentrated under a vacuum at 60°C, and stored at 4°C until usage. The extraction yield of PFP was 10.4% (g/100 g, *w*/*w*). The determination of total polyphenol content in pomegranate polyphenols was performed with a Waters Symmetry Shield RP18 column (250 mm × 4.6 mm × 5 *μ*m) by UV spectrophotometry. Methanol-phosphoric acid solution (5 : 95 *v*/*v*) was used as the mobile phase. The total phenol content in PFP was 40.13% (g/100 g, *w*/*w*). Phenylephrine (PE), acetylcholine (ACh), sodium nitroprusside (SNP), forskolin, tetraethylammonium (TEA), glibenclamide (Glib), BaCl_2_, atropine, and L-NAME were all purchased from Sigma (USA). All the above reagents were dissolved in ultrapure water, except for forskolin and Glib, which were dissolved in dimethyl sulfoxide (DMSO). Ethylene glycol tetraacetic acid (EGTA) and the other chemical reagents were purchased from Chemical Reagents Shanghai Co., Ltd. (Shanghai, China).

### 2.3. Tissue Isolation

Sprague Dawley rats were sacrificed by cervical dislocation. The thoracic aorta was quickly removed and dissected free from connective tissue at room temperature in a Krebs–Henseleit (K-H) solution (118 mM NaCl, 4.7 mM KCl, 25 mM NaHCO_3_, 1.2 mM KH_2_PO_4_, 1.2 mM MgCl_2_, 2.5 mM CaCl_2_, and 2 g/L glucose). The pH of the K-H solution was adjusted to 7.4, following saturation with a 95% O_2_:5% CO_2_ gas mixture.

### 2.4. Relaxation Measurement

Aortic rings (∼3 mm in length) were mounted between two triangle-shaped, stainless-steel hooks in a 20 mL organ bath at 37°C with a 95% O_2_: 5% CO_2_ mixture. The tissues were equilibrated for 60 minutes, and the isotonic, contractile force of the blood vessel was recorded using the BL-420F Biological Function Test System (Taimeng, Chengdu). In order to check the vascular ring activity, the tissues were initially precontracted with 60 mM KCl to get maximum tension under the 1.0 g load for 1 hour before the balance was achieved. The fluid was changed every 15 minutes. Following this, the rings of rat aorta were precontracted with PE (1 *μ*M) for checking the integrity of the vascular endothelium. The endothelium was believed to be intact if 80% or more of the diastolic rate was obtained when treated with 10 *μ*M ACh, while no diastolic response to ACh indicated destruction of the endothelium.

### 2.5. Statistical Analysis

All data were expressed as mean ± standard deviation (*x̅* ± SD). Differences between the groups were analyzed using Student's *t*-test or one-way ANOVA with SPSS 28.0 statistical software (IBM, USA). *PP* values less than 0.05 were considered statistically significant.

## 3. Results and Discussion

### 3.1. PFP Caused Concentration-Dependent Vasodilation with (*E*+) or without (*E−*) Endothelium

**Figure 1:**
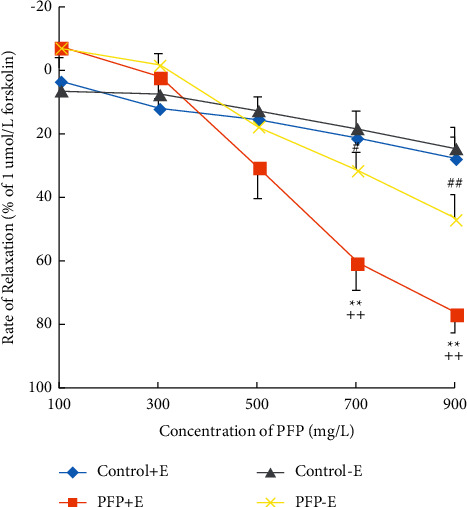
The effect of endothelium on PFP-induced relaxation (*x̅* ± SD, *n* = 6). *Note*. *E*: endothelial; ^*∗∗*^*P* < 0.01 vs. control + *E*;^#^*P* < 0.01 and ^##^*P* < 0.01 vs. control−*E*; and ^++^*P* < 0.01 vs. PFP−*E*.

We investigated the cumulative concentration of PFP (100, 300, 500, 700, and 900 mg/L) on endothelium-intact or endothelium-denuded aortic rings with 1 *µ*M PE. Forskolin (1 *µ*M) was used to obtain maximum dilation of the aortic rings. Relaxation, induced by PFP in the aortic rings, was calculated as a percentage of the relaxation in response to PE. PFP (700 mg/L∼900 mg/L) caused concentration-dependent vasodilation in both endothelium-intact and endothelium-denuded aortic rings. The maximal relaxant effect (*E*_max_) on the PE-induced contraction was 66.84% ± 14.29, while it was 88.78% ± 17.58 for the endothelium-denuded aortic rings (Figure 1).

The ethanol extract of the pomegranate flower is rich in polyphenols, gallic acid, ellagic acid, breviscapus fatty acid ethyl ester, oleanolic acid, ursolic acid, asiatica acid, short-leaf hematoxylic acid ethyl ester, hawthorn acid, carotene, pomegranate, quercetin, and kaempferol [13]. In this study, the relatively higher concentration of PFP (700 mg/L∼900 mg/L) significantly antagonized the constriction caused by PE in normal aortic rings, and this antagonistic effect was more significant in intact arteries than denuded arteries. These results indicated that PFP induced relaxation via both endothelium-dependent and nonendothelium-dependent routes to some extent; vasodilation is partly endothelium-dependent.

### 3.2. Preincubation with L-NAME Significantly Inhibited PFP-Induced Relaxation in Aortic Rings

**Figure 2:**
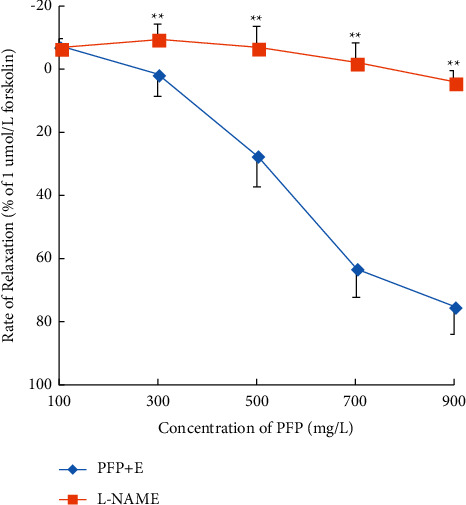
Preincubation with L-NAME significantly inhibited PFP-induced relaxation in aortic rings (*x̅* ± SD, *n* = 6). ^*∗*^*P* < 0.01 and ^*∗∗*^*P* < 0.01 compared to the control group.

Preincubation with L-NAME (10^−4^ mM), a nitric oxide synthase inhibitor, for 30 minutes significantly inhibited the relaxation of endothelium-intact aortic rings that were precontracted by PE treatment. In the presence and absence of L-NAME, Emax was 3.83% ± 3.57% and 74.86% ± 8.90%, respectively (*P* < 0.01; Figure 2). Long-term deficiency of endothelium-derived NO is believed to be either the effect of hyperglycemia-induced endothelial dysfunction or the cause of impairment of vasodilation in diabetes [14, 15]. Preincubation with L-NAME had an inhibitory effect on PFP-induced relaxation in normal aortic rings. Therefore, we speculate that the vasodilator effect of PFP may be related to NO release, which can explain endothelium-dependent vasodilation induced by PFP but cannot explain PFP-induced relaxation on endothelium-denuded aortic rings. Consequently, we went a step further to observe the roles of K^+^ channels and Ca^2+^ in the process.

### 3.3. Preincubation with K^+^ Channel Blockers Significantly Inhibited PFP-Induced Relaxation in Aortic Rings

**Figure 3:**
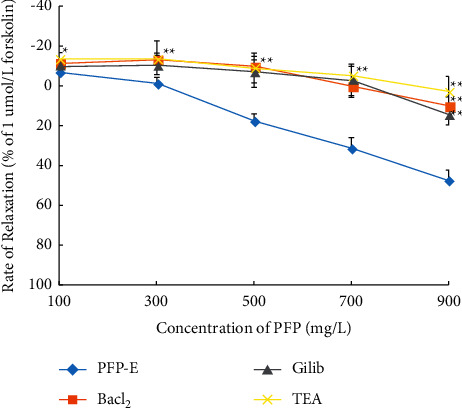
PFP-induced relaxation on denuded aortic rings with or without *K*+ channel inhibitor pretreatment (*x̅* ± SD, *n* = 6). ^*∗*^*P* < 0.01 and ^*∗∗*^*P* < 0.01 compared to the control group.

To determine the possible effects of K^+^ channels on the action of PFP, we tested the vasorelaxant effect of PFP (100, 300, 500, 700, and 900 mg/L) in denuded aortic rings that were preincubated with K^+^ channel blockers: tetraethylammonium (TEA, 3 mM), glibenclamide (10 *µ*M), or BaCl_2_ (100 *µ*mol/L), respectively, for 15 minutes before PE (1 *µ*M) precontraction. The results were calculated as a percentage of the relaxation in response to K^+^ channel blockers' pretreatment on the aortic rings.  Figure 3 shows that the concentration-dependent vasorelaxant effect of PFP was inhibited by K^+^ channel blocker pretreatment. In the PFP (900 mg/L) group, Emax was 47.55% ± 5.17%; in the BaCl_2_ group, Emax was 10.17% ± 4.70%; in the TEA group, Emax was 2.43% ± 7.32%; and in the Glib group, Emax was 14.20% ± 5.32%.

Our findings indicated that TEA (Ca^2+^-activated K^+^ channel blockers), BaCl_2_ (inward rectifying K^+^ channel blockers), and Glib (ATP-sensitive K^+^ channel blockers) significantly inhibit the diastolic effect of PFP on arterial rings, and the inhibitory effect induced by K^+^ channel blockers is very similar. Data indicate that K^+^ channels mediate smooth muscle relaxation in response to NO. The activation of K^+^ channels leads to membrane hyperpolarization, and NO release by eNOS activity subsequently inhibits the opening of voltage-gated Ca^2+^ channels and vasoconstriction [16–18].

### 3.4. PFP Inhibited Both Extracellular Ca^2+^-Induced Contraction and Intracellular Ca^2+^ Releasing Induced by PE

In order to examine the effect of PFP on Ca^2+^ influx, endothelium-denuded aortic rings were incubated in a calcium-free and high potassium (60 mM) K-H solution for 20 minutes, and then a 0.4, 0.8, 1.2, 1.6, 2.0, and 2.4 mM CaCl_2_ solution was added cumulatively to obtain a concentration-response curve and 158 mg/L PFP (EC_50_) was used to pretreat aorta rings for 10 minutes before the application of Ca^2+^. The stable contraction induced by KCl (60 mM) was considered 100%.

To further study the effect of PFP on the release of intracellular Ca^2+^, endothelium-denuded aortic rings were incubated in Ca^2+^-free Krebs–Henseleit solution containing 100 *µ*M EGTA for 10 minutes. PE (1 *µ*M) was added to make the arterial ring continue to precontract slowly and slightly. Subsequently, under the same treatment with the calcium-free K-H solution, the vasoconstriction, induced by PE-stimulated internal calcium release, could be significantly inhibited by PFP (158 mg/L) (*P* < 0.01).

**Figure 4:**
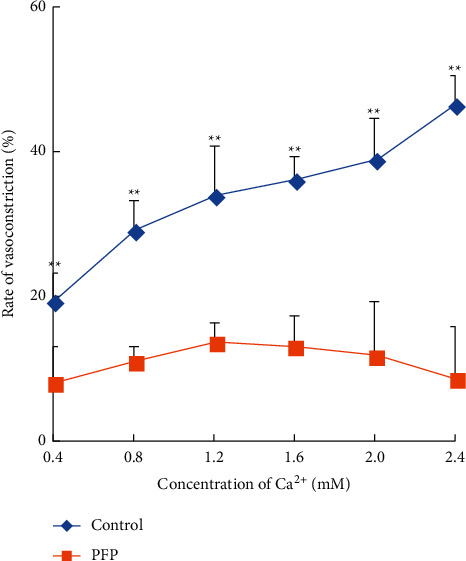
Effect of PFP (158 mg/L) on CaCl_2_ dose-effect curves in calcium-free and high potassium (60 mM) K-H solution in the endothelium-denuded aortic rings (*x̅* ± SD, *n* = 6). ^*∗*^*P* < 0.01 compared to PFP.

As shown in Figure 4, the CaCl_2_ dose-effect curve demonstrates that calcium-induced vasoconstriction (0.4–2.4 mM) was significantly inhibited by PFP in a concentration-dependent manner in calcium-free K-H solution (*P* < 0.01).

**Figure 5:**
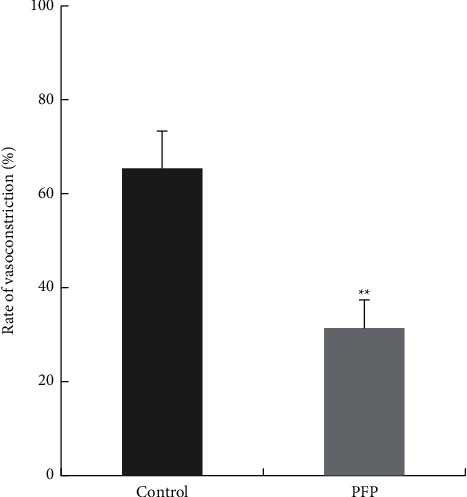
Effect of PFP (158 mg/L) on cytoplasmic calcium release in rat aortic rings with or without endothelium. (*x̅* ± SD, *n* = 6). ^*∗∗*^*P* < 0.01 vs. control.

This possibly indicated that PFP might act on vascular smooth muscle calcium channels and inhibit calcium influx. As shown in Figure 5, the vasoconstriction was significantly inhibited by PFP (*P* < 0.01) in the PFP group, which indicates that the intracellular Ca^2+^ releasing induced by PE could be inhibited by PFP.

Finally, we observed the direct effect of PFP on Ca^2+^ release. The Ca^2+^ ion required for vasoconstriction was partially from the extracellular space through receptor-operated calcium channels (ROCCs) or voltage-dependent calcium channels (VDCCs) in the plasma membrane. It was partially released from the sarcoplasmic reticulum (intracellular Ca^2+^ stores), protein kinase C (PKC) activation, and Ca^2+^ sensitization mechanism [19, 20]. PE induced the influx of extracellular Ca^2+^ by activating ROCCs and KCl-induced Ca^2+^ influx through VDCCs [21]. Our results showed that PFP inhibited Ca^2+^-induced vasoconstriction in the aortic rings, which were precontracted with PE or KCl in Ca^2+^-free KH buffer. These results suggested that PFP significantly inhibited the influx of extracellular Ca^2+^ via ROCCs or VDCCs activated by PE or KCl.

Many compounds, including ellagic acid, gallic acid, oleanolic acid, pomegranate peel pavilion, quercetin, and kaempferol, were isolated from PFP [13]. During the period, the content of tannic acid is among those compounds; ellagic acid is able to induce the release of PLC-dependent Ca^2+^ from the sarcoplasmic reticulum and increase the influx of Ca^2+^ in HepG2 cells [22, 23]. Gallic acid has the ability to induce endothelium-dependent relaxation in rat aortic rings through eNOS, opening potassium channels and the blockade of Ca^2+^ influx via L-type Ca^2+^ channels [24]. It is, therefore, possible that ellagic acid and gallic acid could be responsible for the vasorelaxant effects of PFP. Further investigation is required to examine the exact mechanism of the active compounds of this plant.

### 3.5. A Lower Concentration of PFP Significantly Ameliorates ACh-Mediated Endothelium-Dependent Relaxation Impaired in the Presence of High Glucose

**Figure 6:**
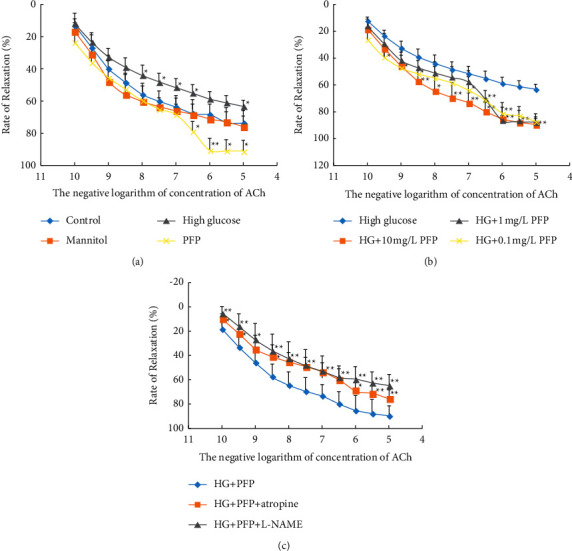
(a) The effect of high glucose and PFP on ACh-mediated relaxation. (b) The effect of different concentrations of PFP incubated with high glucose on ACh-mediated endothelium-dependent relaxation. (c) The effect of atropine and L-NAME on ACh-mediated relaxation of thoracic aortic rings incubated by PFP and high glucose (*x̅* ± SD, *n* = 6). ^*∗*^*P* < 0.01 and ^*∗∗*^*P* < 0.01 vs. control. *Note*. HG: high glucose; HG + PFP: vascular rings were incubated for 6 hours with K-H solution containing 10 mg/L PFP and 44 mM D-glucose; HG + PFP + atropine: vascular rings were pretreated with 0.1 mM atropine for 30 minutes and then incubated with K-H solution containing 10 mg/L PFP and 44 mM D-glucose for 6 hours; HG + PFP + L-NAME: vascular rings were pretreated with 0.1 mM L-NAME for 30 minutes and consequently incubated with K-H solution containing 10 mg/L PFP and 44 mM D-glucose for 6 hours. The diastolic responses mediated by ACh and SNP of thoracic aortic rings in each group were measured.

We subsequently examined the effect of PFP on vasodilation in the presence of high glucose. After 6 hours of incubation with 44 mM glucose, ACh-induced endothelium-dependent vasodilation was significantly reduced: (63.31% ± 3.76%) vs. (73.50% ± 4.15%), (*P* < 0.05). The impaired endothelium-dependent vasorelaxation was significantly restored after 6-hour preincubation with PFP (10 mg/L) (*P* < 0.05) (Figure 6(a)). In the preliminary experiment, the vascular rings lost response to ACh after 6 hours of preincubation with a higher concentration of PFP (100 mg/L and 1000 mg/L) in the presence of high glucose (44 mmol/L). As shown in Figure 6(b), a relatively lower concentration of PFP (0.1～10 mg/L) ameliorates vasorelaxant dysfunction due to hyperglycemia, while PFP in a concentration of 10 mg/L showed a better effect. Therefore, all other experiments were performed with 10 mg/L PFP, and we further investigated the possible mechanism pretreated by atropine and L-NAME on ACh-mediated relaxation of aortic rings incubated by PFP (10 mg/L) and high glucose (44 mmol/L) (Figure 6(c)).

Evidently, the vasorelaxant effect of PFP was inhibited by atropine (*P* < 0.05), as well as L-NAME (nitric oxide synthase inhibitor), in the presence of high glucose (*P* < 0.05). In this study, L-NAME had an inhibitory effect on PFP-induced relaxation with or without high glucose but had no significant effect on denuded arteries (Table 1 and Figure 6(c)). This is consistent with the finding of Rocha-Resende et al. that increased local cholinergic activity leads to increased NO levels [25]. A possible explanation for those results was that PFP might promote endothelium-dependent vasodilation against high glucose via a cholinergic pathway, which subsequently increased NO production. This speculation was further confirmed by NO content measurement in the blood vessel by the Griess method (Table 2). The beneficial activities of NO in the vascular system, by regulating eNOS and nNOS signaling pathways, have already been discussed by a series of studies. Type 2 diabetes and cardiovascular disease are characterized by poor control of the endothelial cell redox environment, with a shift toward the overproduction of reactive oxygen species (ROS) by nicotinamide adenine dinucleotide phosphate oxidase (NOX) [25–27].

### 3.6. PFP and High Glucose Did Not Affect the SNP-Mediated Nonendothelium-Dependent Relaxation

**Figure 7:**
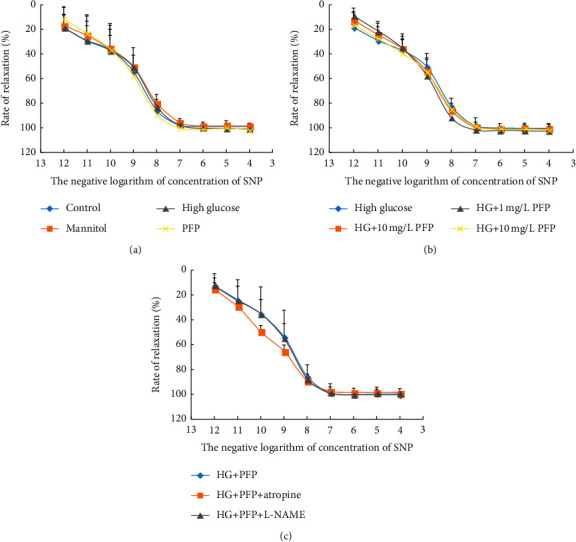
(a) The effect of high glucose and PFP on nonendothelium-dependent relaxation of aortic rings mediated by SNP. (b) The effect of different concentrations of PFP on nonendothelium-dependent relaxation of aorta rings induced by SNP-mediated high glucose. (c) The effect of atropine and L-NAME on SNP-mediated endothelium-dependent relaxation incubated with PFP and high glucose (x̅ ± SD, *n* = 6). *Note*. HG: high glucose; HG + PFP: vascular rings were incubated for 6 hours with K-H solution containing 10 mg/L PFP and 44 mM D-glucose; HG + PFP + atropine: vascular rings were pretreated with 0.1 mM atropine for 30 minutes and then incubated with K-H solution containing 10 mg/L PFP and 44 mM D-glucose for 6 hours; HG + PFP + L-NAME: vascular rings were pretreated with 0.1 mM L-NAME for 30 minutes and then incubated with K-H solution containing 10 mg/L PFP and 44 mM D-glucose for 6 hours. The diastolic responses mediated by ACh and SNP of the thoracic aortic rings in each group were measured.

To evaluate the action of PFP on nonendothelium-dependent vasodilation in the presence of high glucose, aortic rings were incubated in different glucose concentrations (11 mmol/L and 44 mmol/L) or hypertonic K-H solution for 6 hours. After the arterial ring was stabilized, PEs of 1 *µ*mol/L were added to the bath and SNP (10^−12^ mol/L, 10^−11^ mol/L, 10^−10^ mol/L, 10^−9^ mol/L, 10^−8^ mol/L, and 10^−7^ mol/L) was gradually added after the peak contraction. The first dose was balanced, and then the next dose was given. The diastolic response of thoracic aortic rings to SNP was measured. The diastolic rate of arterial rings was expressed by the ratio between the diastolic amplitude of the arteries after SNP injection and the maximum contractile amplitude induced by PE. A concentration-diastolic curve of an SNP was drawn. The difference between groups has no statistical significance in Figures 7(a)–7(c); Emax values are given in Table 3. Atropine and L-NAME did not affect the nonendothelium-dependent relaxation of aortic rings induced by SNP-mediated PFP and high glucose in rats (Figure 7(c)).

In the present study, high glucose preincubation had little effect on the NO donor (SNP) mediated endothelium-independent relaxation. Considering that vasodiastolic effects of SNP are independent of the integrity of vascular intima [28], the result suggested that high glucose might not damage the smooth muscle function in the aortic ring. Different from the vasodilation of higher-concentration PFP in normal aortic rings, lower-concentration PFP (0.1–10 mg/L) significantly ameliorated impaired vascular endothelium-dependent relaxation that was induced by high glucose. However, it had no effect on nonendothelium-dependent relaxation in the presence of high glucose.

Moreover, studies showed that chronic and acute hyperglycemia increases the production of ROS, resulting in the development of oxidative stress and endothelial dysfunction [29]. ROS-induced vascular dysfunction occurs through the impairment of endothelial-derived relaxing factors such as NO as well as endothelium-derived hyperpolarization [28]. Therefore, one of the mechanisms by which PFP ameliorates vasodilation is presumably related to its antioxidant capacity *in vivo* and *in vitro* [11, 30–32]. As to why different concentrations of PFP show different diastolic effects, with or without high glucose, we need more research on it in the future. In previous experiments with rats, the effective dose of PFP was 100 mg/kg and 150 mg/kg [3, 11, 30, 32–34]. A low concentration of PFP (0.1–10 mg/L) is inactive on the aorta in the absence of high glucose levels, which may be because the concentration of PFP is at a theoretically safer level.

### 3.7. NO Content in Vascular Tissue

The NO content in the aortic vascular tissue supernatant was determined according to the manufacturer's instructions. The content of NO in vascular tissues of the high glucose treatment group was significantly lower than that of the control group (*P* < 0.01), while the content of NO in vascular tissues of the mannitol group did not change significantly (*P* < 0.05). PFP treatment did not significantly change the NO content in vascular tissue (*P* < 0.05). Compared with the high glucose group, the NO content in the vascular tissues of the high glucose group treated with PFP (10 mg/L) was significantly increased (*P* < 0.01) (Table 3).

## 4. Conclusions

Oxidative stress caused by diabetes mellitus has detrimental effects on blood vessels, leading to impaired diastole. Therefore, in addition to treating diabetes, the use of antioxidants to reduce the negative effects of oxidative stress has many therapeutic benefits. The results of this study showed the ability of PFP antioxidants to reduce the effect of oxidative stress and significantly improve diastolic relaxation in rats with hyperglycemia. Thus, we recommend the use of PFP as a dietary supplement for diabetic patients with hypertension. We would like to indicate the need for more studies to fully understand the mechanism of this effect, especially on humans.

## Figures and Tables

**Table 1:** ACh-mediated and SNP-mediated endothelium-dependent relaxation in response to different concentrations of PFP incubated with high glucose (*x̅* ± SD, *n* = 6).

Group	*E*_max_ (%) ACh	*E*_max_ (%) SNP
High glucose + PFP	89.23 ± 7.88	100.78 ± 0.93
High glucose + PFP + atropine	75.11 ± 6.87^∗∗^	99.14 ± 3.63
High glucose + PFP + L-NAME	64.41 ± 9.03^∗∗^	99.49 ± 3.87

**Table 2:** Nitric oxide content in blood vessels in each group (*x̅* ± SD, *n* = 6).

Group	NO (mmol/g)
Control	4.59 ± 0.64
High glucose	2.99 ± 0.78^∗∗^
Mannitol	4.24 ± 0.68
10 mg/L PFP	4.37 ± 0.58
High glucose + 10 mg/L PFP	4.10 ± 0.27^##^
High glucose + 1 mg/L PFP	3.52 ± 0.57
High glucose + 0.1 mg/L PFP	3.30 ± 0.46

**Table 3:** ACh-mediated and SNP-mediated endothelium-dependent relaxation in response to different PFP concentrations incubated with high glucose (*x̅* ± SD, *n* = 6).

Group	*E*_max_ (%) ACh	*E*_max_ (%) SNP
High glucose	86.96 ± 3.31^∗∗^	101.18 ± 2.32
High glucose + 10 mg/L PFP	63.31 ± 3.76	100.78 ± 0.93
High glucose + 1 mg/L PFP	89.23 ± 7.88^∗∗^	102.80 ± 5.50
High glucose + 0.1 mg/L PFP	87.67 ± 2.98^∗∗^	102.15 ± 5.80

## Data Availability

The data supporting the findings of this study are available within the article.
